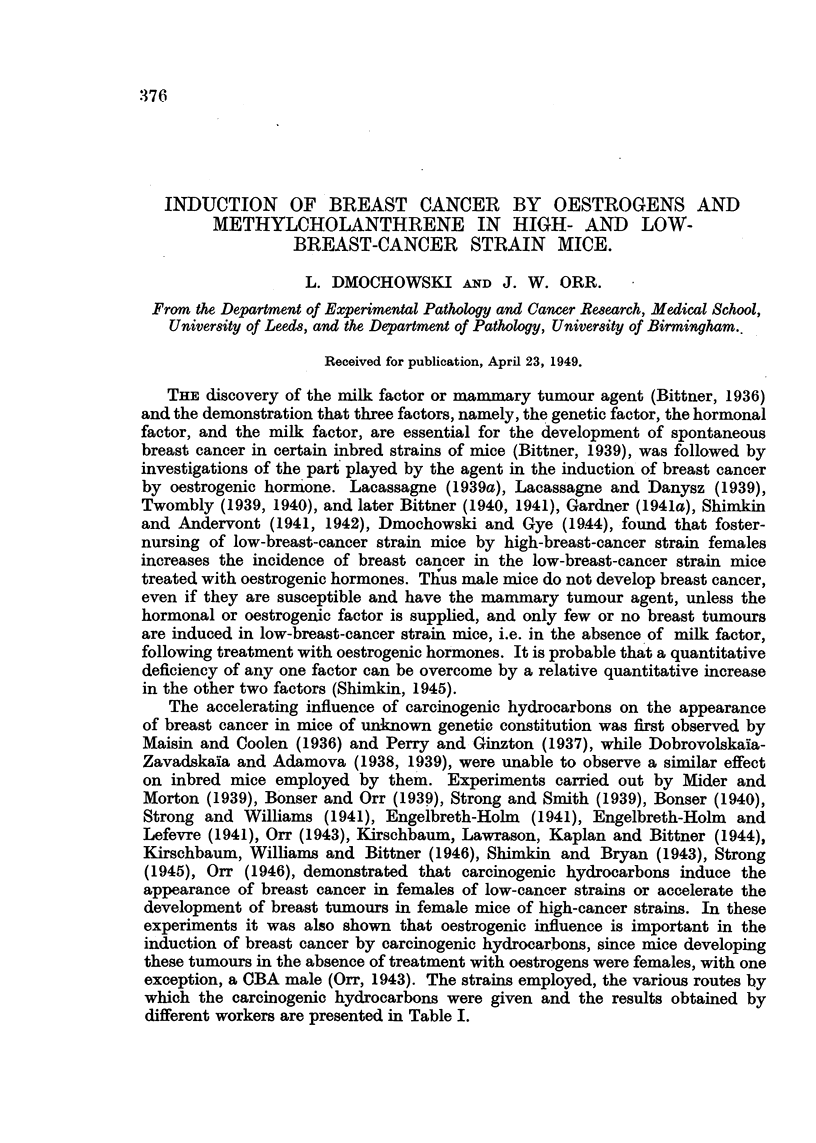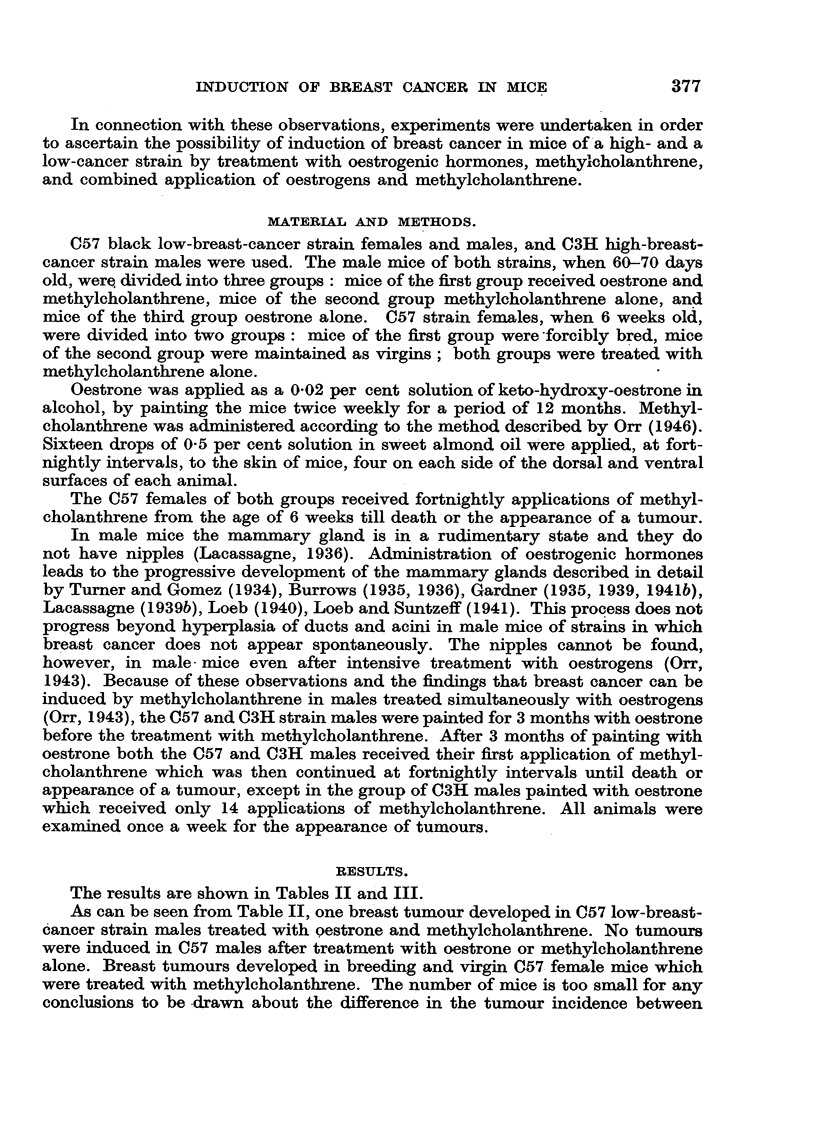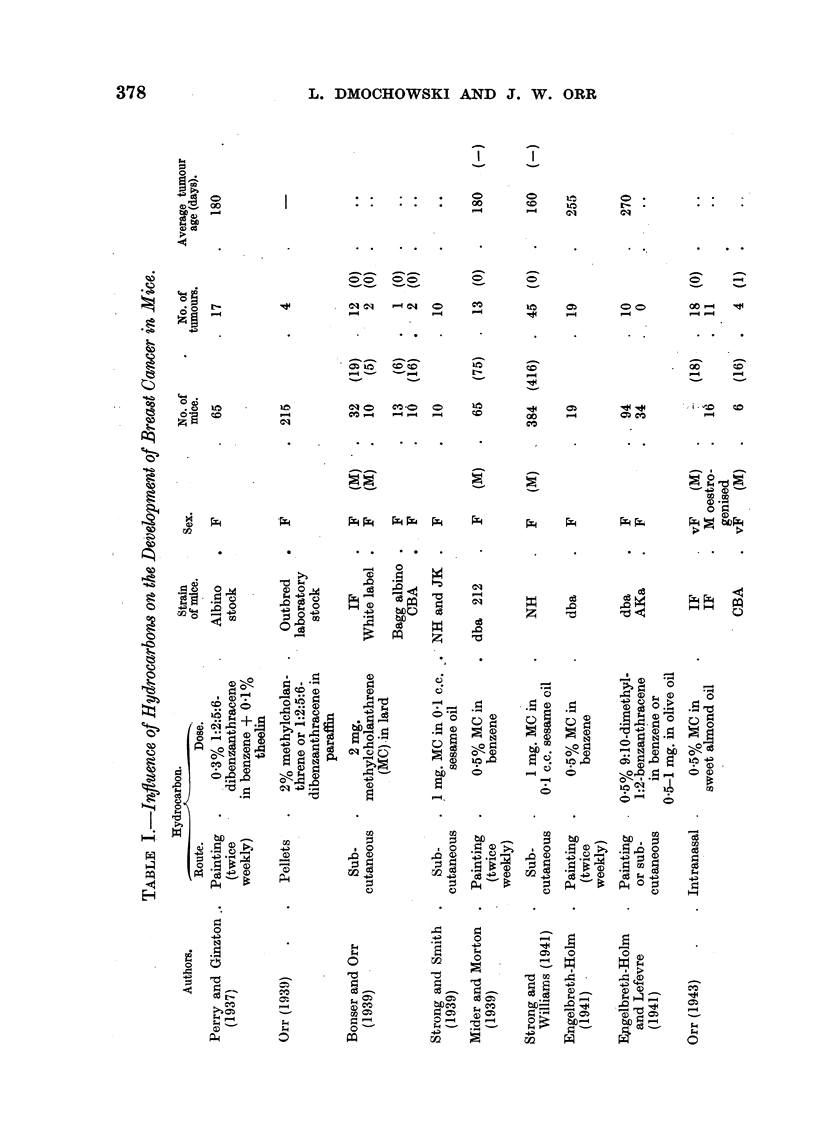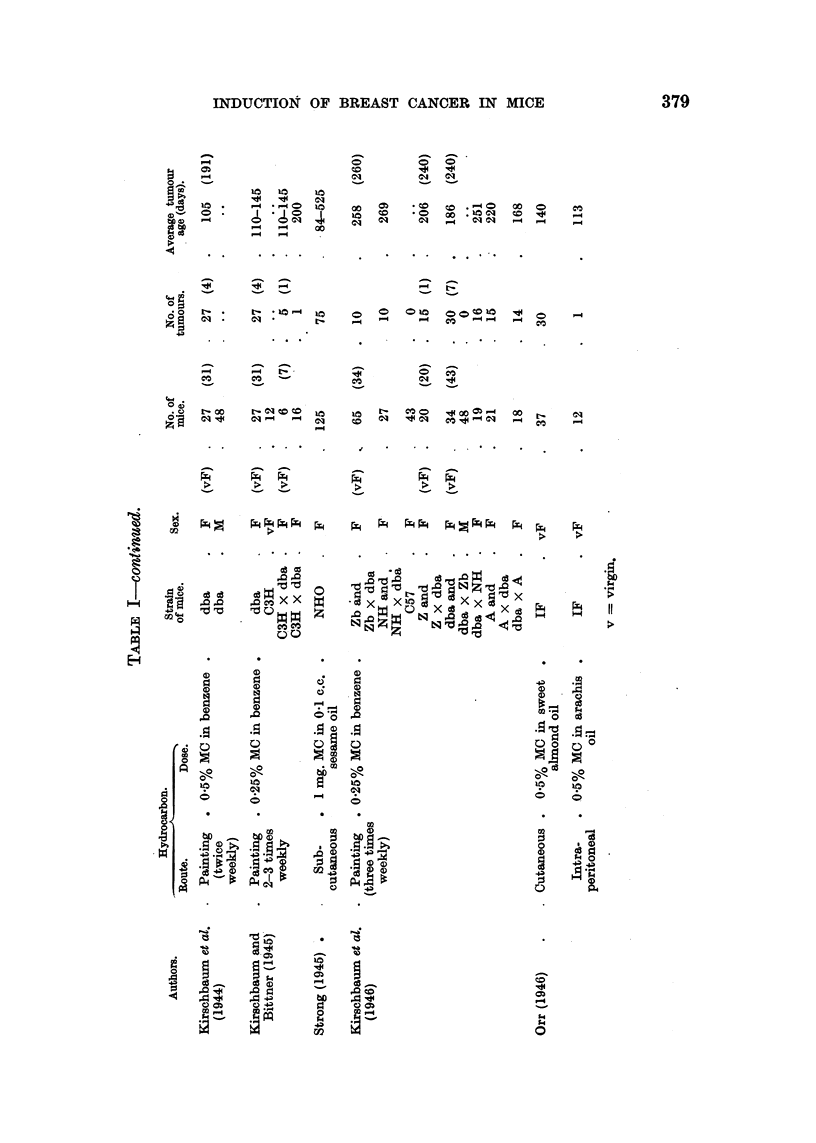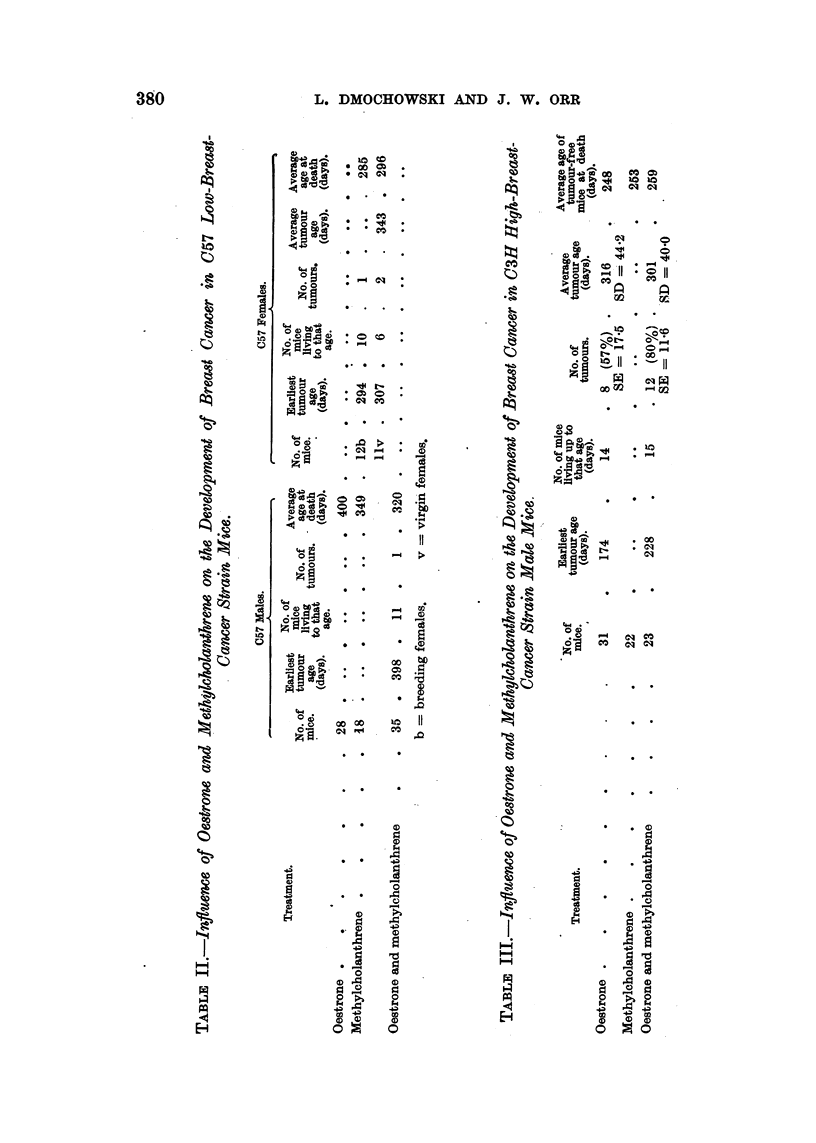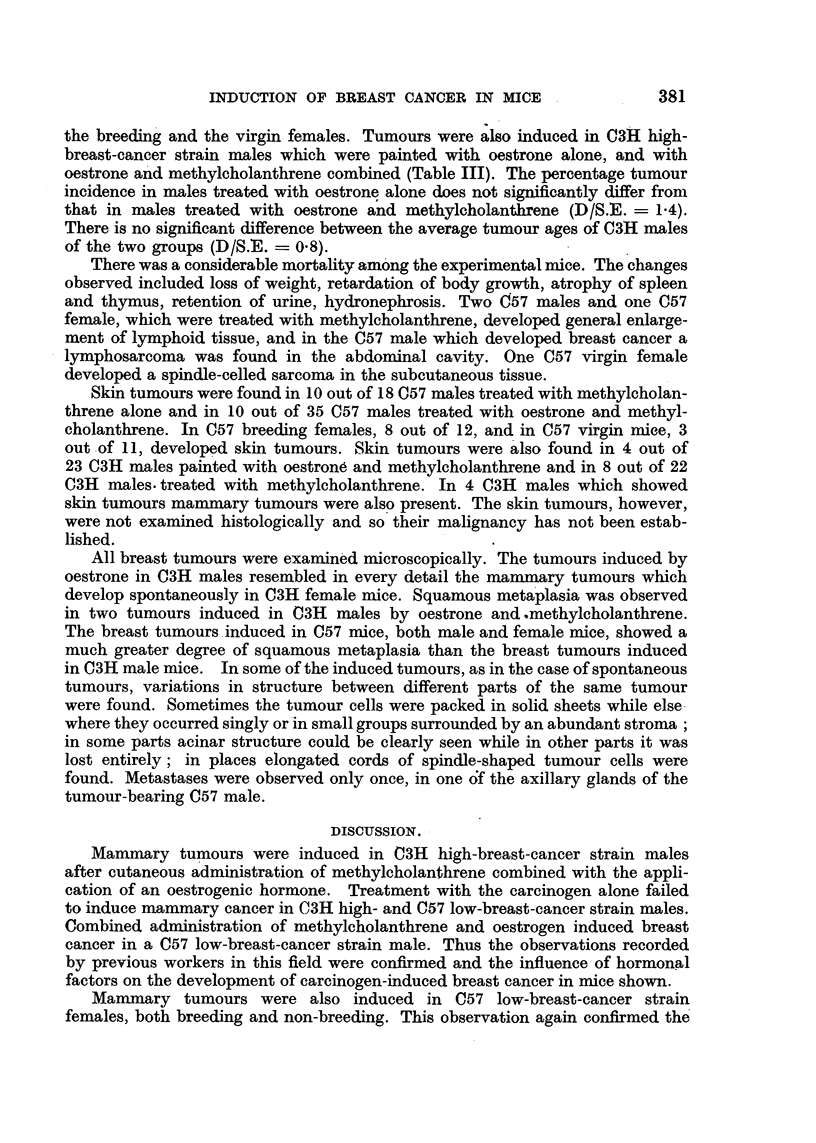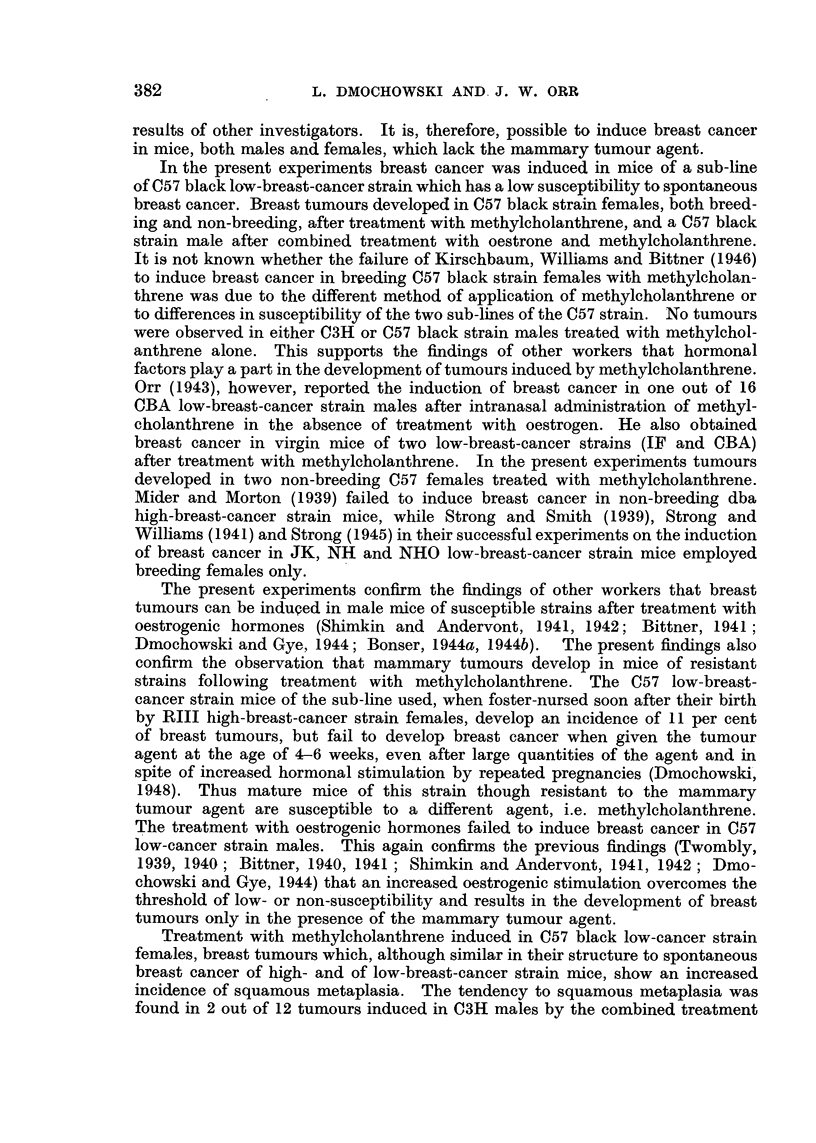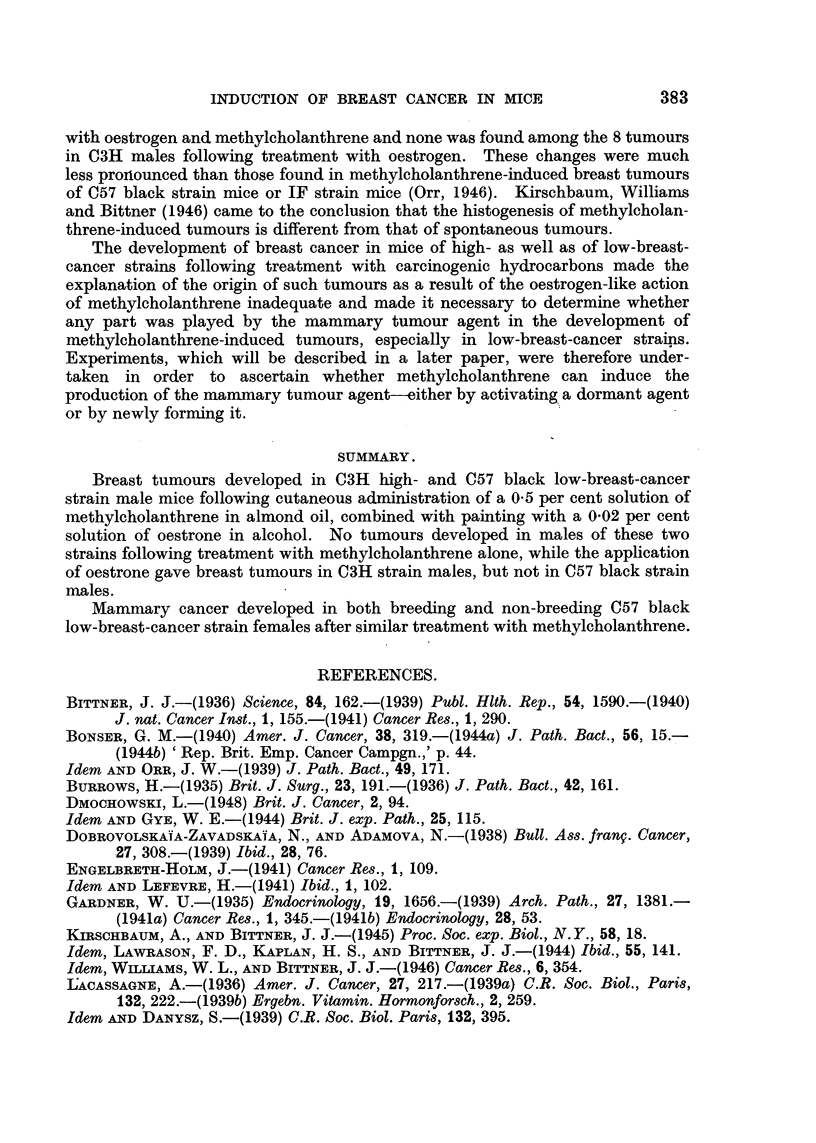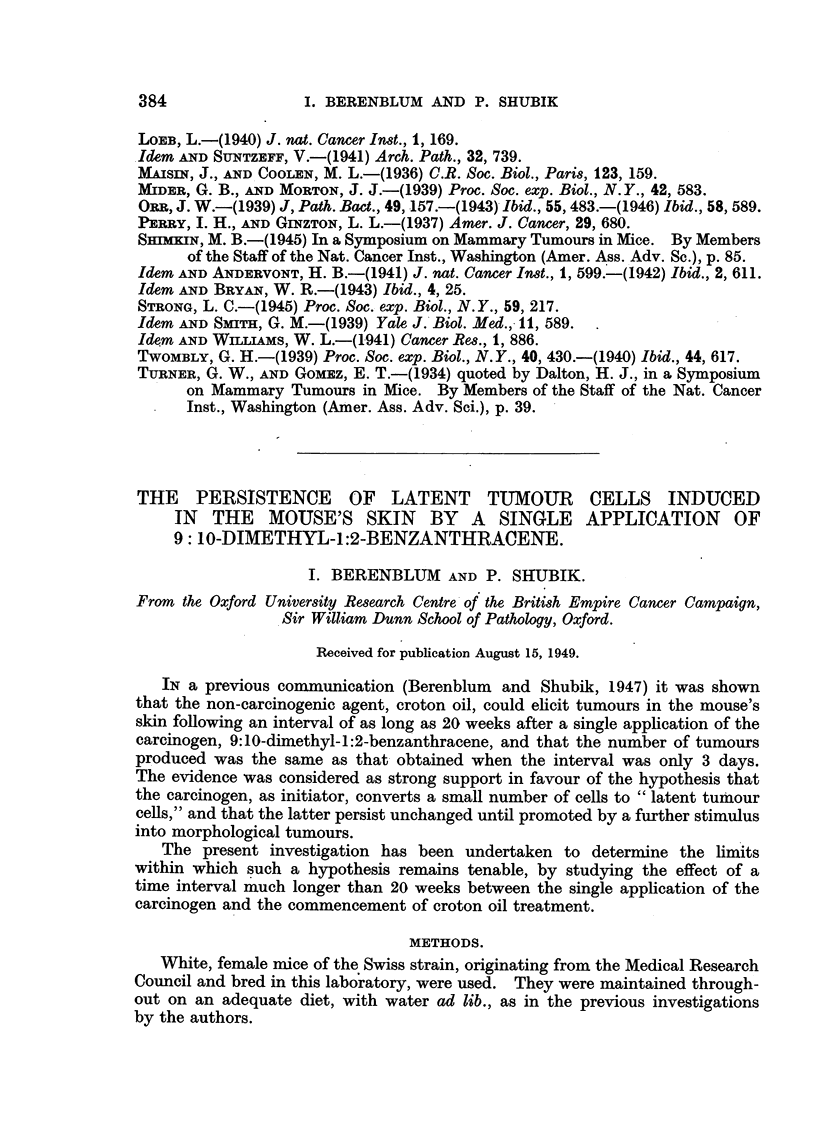# Induction of Breast Cancer by Oestrogens and Methylcholanthrene in High- and Low-Breast-Cancer Strain Mice

**DOI:** 10.1038/bjc.1949.41

**Published:** 1949-09

**Authors:** L. Dmochowski, J. W. Orr


### INDUCTION OF BREAST CANCER BY OESTROGENS AND METHYLCHOLANTHRENE IN HIGH- AND LOW-BREAST-CANCER STRAIN MICE.

L. DMOCHOWSKI AND J. W. ORR.

*From the Department of Experimental Pathology and Cancer Research, Medical School, University of Leeds, and the Department of Pathology, University of Birmingham.*

Received for publication, April 23, 1949.

THE discovery of the milk factor or mammary tumour agent (Bittner, 1936) and the demonstration that three factors, namely, the genetic factor, the hormonal factor, and the milk factor, are essential for the development of spontaneous breast cancer in certain inbred strains of mice (Bittner, 1939), was followed by investigations of the part played by the agent in the induction of breast cancer by oestrogenic hormone. Lacassagne (1939*a*), Lacassagne and Danysz (1939), Twombly (1939, 1940), and later Bittner (1940, 1941), Gardner (1941*a*), Shimkin and Andervont (1941, 1942), Dmochowski and Gye (1944), found that foster-nursing of low-breast-cancer strain mice by high-breast-cancer strain females increases the incidence of breast cancer in the low-breast-cancer strain mice treated with oestrogenic hormones. Thus male mice do not develop breast cancer, even if they are susceptible and have the mammary tumour agent, unless the hormonal or oestrogenic factor is supplied, and only few or no breast tumours are induced in low-breast-cancer strain mice, i.e. in the absence of milk factor, following treatment with oestrogenic hormones. It is probable that a quantitative deficiency of any one factor can be overcome by a relative quantitative increase in the other two factors (Shimkin, 1945).

The accelerating influence of carcinogenic hydrocarbons on the appearance of breast cancer in mice of unknown genetic constitution was first observed by Maisin and Coolen (1936) and Perry and Ginzton (1937), while Dobrovolskaïa-Zavadskaïa and Adamova (1938, 1939), were unable to observe a similar effect on inbred mice employed by them. Experiments carried out by Mider and Morton (1939), Bonser and Orr (1939), Strong and Smith (1939), Bonser (1940), Strong and Williams (1941), Engelbreth-Holm (1941), Engelbreth-Holm and Lefevre (1941), Orr (1943), Kirschbaum, Lawrason, Kaplan and Bittner (1944), Kirschbaum, Williams and Bittner (1946), Shimkin and Bryan (1943), Strong (1945), Orr (1946), demonstrated that carcinogenic hydrocarbons induce the appearance of breast cancer in females of low-cancer strains or accelerate the development of breast tumours in female mice of high-cancer strains. In these experiments it was also shown that oestrogenic influence is important in the induction of breast cancer by carcinogenic hydrocarbons, since mice developing these tumours in the absence of treatment with oestrogens were females, with one exception, a CBA male (Orr, 1943). The strains employed, the various routes by which the carcinogenic hydrocarbons were given and the results obtained by different workers are presented in Table I.

In connection with these observations, experiments were undertaken in order to ascertain the possibility of induction of breast cancer in mice of a high- and a low-cancer strain by treatment with oestrogenic hormones, methylcholanthrene, and combined application of oestrogens and methylcholanthrene.

### MATERIAL AND METHODS.

C57 black low-breast-cancer strain females and males, and C3H high-breast-cancer strain males were used. The male mice of both strains, when 60–70 days old, were divided into three groups : mice of the first group received oestrone and methylcholanthrene, mice of the second group methylcholanthrene alone, and mice of the third group oestrone alone. C57 strain females, when 6 weeks old, were divided into two groups : mice of the first group were forcibly bred, mice of the second group were maintained as virgins ; both groups were treated with methylcholanthrene alone.

Oestrone was applied as a 0·02 per cent solution of keto-hydroxy-oestrone in alcohol, by painting the mice twice weekly for a period of 12 months. Methylcholanthrene was administered according to the method described by Orr (1946). Sixteen drops of 0·5 per cent solution in sweet almond oil were applied, at fortnightly intervals, to the skin of mice, four on each side of the dorsal and ventral surfaces of each animal.

The C57 females of both groups received fortnightly applications of methylcholanthrene from the age of 6 weeks till death or the appearance of a tumour.

In male mice the mammary gland is in a rudimentary state and they do not have nipples (Lacassagne, 1936). Administration of oestrogenic hormones leads to the progressive development of the mammary glands described in detail by Turner and Gomez (1934), Burrows (1935, 1936), Gardner (1935, 1939, 1941*b*), Lacassagne (1939*b*), Loeb (1940), Loeb and Suntzeff (1941). This process does not progress beyond hyperplasia of ducts and acini in male mice of strains in which breast cancer does not appear spontaneously. The nipples cannot be found, however, in male mice even after intensive treatment with oestrogens (Orr, 1943). Because of these observations and the findings that breast cancer can be induced by methylcholanthrene in males treated simultaneously with oestrogens (Orr, 1943), the C57 and C3H strain males were painted for 3 months with oestrone before the treatment with methylcholanthrene. After 3 months of painting with oestrone both the C57 and C3H males received their first application of methylcholanthrene which was then continued at fortnightly intervals until death or appearance of a tumour, except in the group of C3H males painted with oestrone which received only 14 applications of methylcholanthrene. All animals were examined once a week for the appearance of tumours.

### RESULTS.

The results are shown in Tables II and III.

As can be seen from Table II, one breast tumour developed in C57 low-breast-cancer strain males treated with oestrone and methylcholanthrene. No tumours were induced in C57 males after treatment with oestrone or methylcholanthrene alone. Breast tumours developed in breeding and virgin C57 female mice which were treated with methylcholanthrene. The number of mice is too small for any conclusions to be drawn about the difference in the tumour incidence between the breeding and the virgin females. Tumours were also induced in C3H high-breast-cancer strain males which were painted with oestrone alone, and with oestrone and methylcholanthrene combined (Table III). The percentage tumour incidence in males treated with oestrone alone does not significantly differ from that in males treated with oestrone and methylcholanthrene (D/S.E. = 1·4). There is no significant difference between the average tumour ages of C3H males of the two groups (D/S.E. = 0·8).

There was a considerable mortality among the experimental mice. The changes observed included loss of weight, retardation of body growth, atrophy of spleen and thymus, retention of urine, hydronephrosis. Two C57 males and one C57 female, which were treated with methylcholanthrene, developed general enlargement of lymphoid tissue, and in the C57 male which developed breast cancer a lymphosarcoma was found in the abdominal cavity. One C57 virgin female developed a spindle-celled sarcoma in the subcutaneous tissue.

Skin tumours were found in 10 out of 18 C57 males treated with methylcholanthrene alone and in 10 out of 35 C57 males treated with oestrone and methylcholanthrene. In C57 breeding females, 8 out of 12, and in C57 virgin mice, 3 out of 11, developed skin tumours. Skin tumours were also found in 4 out of 23 C3H males painted with oestrone and methylcholanthrene and in 8 out of 22 C3H males treated with methylcholanthrene. In 4 C3H males which showed skin tumours mammary tumours were also present. The skin tumours, however, were not examined histologically and so their malignancy has not been established.

All breast tumours were examined microscopically. The tumours induced by oestrone in C3H males resembled in every detail the mammary tumours which develop spontaneously in C3H female mice. Squamous metaplasia was observed in two tumours induced in C3H males by oestrone and methylcholanthrene. The breast tumours induced in C57 mice, both male and female mice, showed a much greater degree of squamous metaplasia than the breast tumours induced in C3H male mice. In some of the induced tumours, as in the case of spontaneous tumours, variations in structure between different parts of the same tumour were found. Sometimes the tumour cells were packed in solid sheets while elsewhere they occurred singly or in small groups surrounded by an abundant stroma; in some parts acinar structure could be clearly seen while in other parts it was lost entirely; in places elongated cords of spindle-shaped tumour cells were found. Metastases were observed only once, in one of the axillary glands of the tumour-bearing C57 male.

### DISCUSSION.

Mammary tumours were induced in C3H high-breast-cancer strain males after cutaneous administration of methylcholanthrene combined with the application of an oestrogenic hormone. Treatment with the carcinogen alone failed to induce mammary cancer in C3H high- and C57 low-breast-cancer strain males. Combined administration of methylcholanthrene and oestrogen induced breast cancer in a C57 low-breast-cancer strain male. Thus the observations recorded by previous workers in this field were confirmed and the influence of hormonal factors on the development of carcinogen-induced breast cancer in mice shown.

Mammary tumours were also induced in C57 low-breast-cancer strain females, both breeding and non-breeding. This observation again confirmed the results of other investigators. It is, therefore, possible to induce breast cancer in mice, both males and females, which lack the mammary tumour agent.

In the present experiments breast cancer was induced in mice of a sub-line of C57 black low-breast-cancer strain which has a low susceptibility to spontaneous breast cancer. Breast tumours developed in C57 black strain females, both breeding and non-breeding, after treatment with methylcholanthrene, and a C57 black strain male after combined treatment with oestrone and methylcholanthrene. It is not known whether the failure of Kirschbaum, Williams and Bittner (1946) to induce breast cancer in breeding C57 black strain females with methylcholanthrene was due to the different method of application of methylcholanthrene or to differences in susceptibility of the two sub-lines of the C57 strain. No tumours were observed in either C3H or C57 black strain males treated with methylcholanthrene alone. This supports the findings of other workers that hormonal factors play a part in the development of tumours induced by methylcholanthrene. Orr (1943), however, reported the induction of breast cancer in one out of 16 CBA low-breast-cancer strain males after intranasal administration of methylcholanthrene in the absence of treatment with oestrogen. He also obtained breast cancer in virgin mice of two low-breast-cancer strains (IF and CBA) after treatment with methylcholanthrene. In the present experiments tumours developed in two non-breeding C57 females treated with methylcholanthrene. Mider and Morton (1939) failed to induce breast cancer in non-breeding dba high-breast-cancer strain mice, while Strong and Smith (1939), Strong and Williams (1941) and Strong (1945) in their successful experiments on the induction of breast cancer in JK, NH and NHO low-breast-cancer strain mice employed breeding females only.

The present experiments confirm the findings of other workers that breast tumours can be induced in male mice of susceptible strains after treatment with oestrogenic hormones (Shimkin and Andervont, 1941, 1942; Bittner, 1941; Dmochowski and Gye, 1944; Bonser, 1944*a*, 1944*b*). The present findings also confirm the observation that mammary tumours develop in mice of resistant strains following treatment with methylcholanthrene. The C57 low-breast-cancer strain mice of the sub-line used, when foster-nursed soon after their birth by RIII high-breast-cancer strain females, develop an incidence of 11 per cent of breast tumours, but fail to develop breast cancer when given the tumour agent at the age of 4–6 weeks, even after large quantities of the agent and in spite of increased hormonal stimulation by repeated pregnancies (Dmochowski, 1948). Thus mature mice of this strain though resistant to the mammary tumour agent are susceptible to a different agent, i.e. methylcholanthrene. The treatment with oestrogenic hormones failed to induce breast cancer in C57 low-cancer strain males. This again confirms the previous findings (Twombly, 1939, 1940; Bittner, 1940, 1941; Shimkin and Andervont, 1941, 1942; Dmochowski and Gye, 1944) that an increased oestrogenic stimulation overcomes the threshold of low- or non-susceptibility and results in the development of breast tumours only in the presence of the mammary tumour agent.

Treatment with methylcholanthrene induced in C57 black low-cancer strain females, breast tumours which, although similar in their structure to spontaneous breast cancer of high- and of low-breast-cancer strain mice, show an increased incidence of squamous metaplasia. The tendency to squamous metaplasia was found in 2 out of 12 tumours induced in C3H males by the combined treatment with oestrogen and methylcholanthrene and none was found among the 8 tumours in C3H males following treatment with oestrogen. These changes were much less pronounced than those found in methylcholanthrene-induced breast tumours of C57 black strain mice or IF strain mice (Orr, 1946). Kirschbaum, Williams and Bittner (1946) came to the conclusion that the histogenesis of methylcholanthrene-induced tumours is different from that of spontaneous tumours.

The development of breast cancer in mice of high- as well as of low-breast-cancer strains following treatment with carcinogenic hydrocarbons made the explanation of the origin of such tumours as a result of the oestrogen-like action of methylcholanthrene inadequate and made it necessary to determine whether any part was played by the mammary tumour agent in the development of methylcholanthrene-induced tumours, especially in low-breast-cancer strains. Experiments, which will be described in a later paper, were therefore undertaken in order to ascertain whether methylcholanthrene can induce the production of the mammary tumour agent—either by activating a dormant agent or by newly forming it.

### SUMMARY.

Breast tumours developed in C3H high- and C57 black low-breast-cancer strain male mice following cutaneous administration of a 0·5 per cent solution of methylcholanthrene in almond oil, combined with painting with a 0·02 per cent solution of oestrone in alcohol. No tumours developed in males of these two strains following treatment with methylcholanthrene alone, while the application of oestrone gave breast tumours in C3H strain males, but not in C57 black strain males.

Mammary cancer developed in both breeding and non-breeding C57 black low-breast-cancer strain females after similar treatment with methylcholanthrene.

### REFERENCES.

Bittner, J. J.—(1936) *Science*, **84**, 162.—(1939) *Publ. Hlth. Rep.*, **54**, 1590.—(1940) *J. nat. Cancer Inst.*, **1**, 155.—(1941) *Cancer Res.*, **1**, 290.

Bonser, G. M.—(1940) *Amer. J. Cancer*, **38**, 319.—(1944*a*) *J. Path. Bact.*, **56**, 15.—(1944*b*) ' Rep. Brit. Emp. Cancer Campgn.,' p. 44.

*Idem* and Orr, J. W.—(1939) *J. Path. Bact.*, **49**, 171.

Burrows, H.—(1935) *Brit. J. Surg.*, **23**, 191.—(1936) *J. Path. Bact.*, **42**, 161.

Dmochowski, L.—(1948) *Brit. J. Cancer*, **2**, 94.

*Idem* and Gye, W. E.—(1944) *Brit. J. exp. Path.*, **25**, 115.

Dobrovolskaïa-Zavadskaïa, N., and Adamova, N.—(1938) *Bull. Ass. franç. Cancer*, **27**, 308.—(1939) *Ibid.*, **28**, 76.

Engelbreth-Holm, J.—(1941) *Cancer Res.*, **1**, 109.

*Idem* and Lefevre, H.—(1941) *Ibid.*, **1**, 102.

Gardner, W. U.—(1935) *Endocrinology*, **19**, 1656.—(1939) *Arch. Path.*, **27**, 1381.—(1941*a*) *Cancer Res.*, **1**, 345.—(1941*b*) *Endocrinology*, **28**, 53.

Kirschbaum, A., and Bittner, J. J.—(1945) *Proc. Soc. exp. Biol., N.Y.*, **58**, 18.

*Idem*, Lawrason, F. D., Kaplan, H. S., and Bittner, J. J.—(1944) *Ibid.*, **55**, 141.

*Idem*, Williams, W. L., and Bittner, J. J.—(1946) *Cancer Res.*, **6**, 354.

Lacassagne, A.—(1936) *Amer. J. Cancer*, **27**, 217.—(1939*a*) *C.R. Soc. Biol., Paris*, **132**, 222.—(1939*b*) *Ergebn. Vitamin. Hormonforsch.*, **2**, 259.

*Idem* and Danysz, S.—(1939) *C.R. Soc. Biol. Paris*, **132**, 395.

LOEB, L.—(1940) *J. nat. Cancer Inst.*, **1**, 169.
*Idem* AND SUNTZEFF, V.—(1941) *Arch. Path.*, **32**, 739.
MAISIN, J., AND COOLEN, M. L.—(1936) *C.R. Soc. Biol., Paris*, **123**, 159.
MIDER, G. B., AND MORTON, J. J.—(1939) *Proc. Soc. exp. Biol., N.Y.*, **42**, 583.
ORR, J. W.—(1939) *J, Path. Bact.*, **49**, 157.—(1943) *Ibid.*, **55**, 483.—(1946) *Ibid.*, **58**, 589.
PERRY, I. H., AND GINZTON, L. L.—(1937) *Amer. J. Cancer*, **29**, 680.
SHIMKIN, M. B.—(1945) In a Symposium on Mammary Tumours in Mice. By Members of the Staff of the Nat. Cancer Inst., Washington (Amer. Ass. Adv. Sc.), p. 85.
*Idem* AND ANDERVONT, H. B.—(1941) *J. nat. Cancer Inst.*, **1**, 599.—(1942) *Ibid.*, **2**, 611.
*Idem* AND BRYAN, W. R.—(1943) *Ibid.*, **4**, 25.
STRONG, L. C.—(1945) *Proc. Soc. exp. Biol., N.Y.*, **59**, 217.
*Idem* AND SMITH, G. M.—(1939) *Yale J. Biol. Med.*, **11**, 589.
*Idem* AND WILLIAMS, W. L.—(1941) *Cancer Res.*, **1**, 886.
TWOMBLY, G. H.—(1939) *Proc. Soc. exp. Biol., N.Y.*, **40**, 430.—(1940) *Ibid.*, **44**, 617.
TURNER, G. W., AND GOMEZ, E. T.—(1934) quoted by Dalton, H. J., in a Symposium on Mammary Tumours in Mice. By Members of the Staff of the Nat. Cancer Inst., Washington (Amer. Ass. Adv. Sci.), p. 39.

---

### THE PERSISTENCE OF LATENT TUMOUR CELLS INDUCED IN THE MOUSE'S SKIN BY A SINGLE APPLICATION OF 9:10-DIMETHYL-1:2-BENZANTHRACENE.

I. BERENBLUM AND P. SHUBIK.